# Socio-economic predictors of stillbirths in Nepal (2001-2011)

**DOI:** 10.1371/journal.pone.0181332

**Published:** 2017-07-13

**Authors:** Pramesh Raj Ghimire, Kingsley Emwinyore Agho, Andre Renzaho, Aliki Christou, Monjura Khatun Nisha, Michael Dibley, Camille Raynes-Greenow

**Affiliations:** 1 School of Science and Health, Western Sydney University, Penrith, New South Wales, Australia; 2 School of Social Sciences and Psychology, Western Sydney University, Penrith, New South Wales, Australia; 3 Sydney School of Public Health, University of Sydney, Sydney, New South Wales, Australia; National Academy of Medical Sciences, NEPAL

## Abstract

**Introduction:**

Stillbirth has a long-lasting impact on parents and families. This study examined socio-economic predictors associated with stillbirth in Nepal for the year 2001, 2006 and 2011.

**Methods:**

The Nepalese Demographic and Health Survey (NDHS) data for the period (2001–2011) were pooled to estimate socio-economic predictors associated with stillbirths in Nepal using binomial logistic regression while taking clustering and sampling weights into account.

**Results:**

A total of 18,386 pregnancies of at least 28 weeks gestation were identified. Of these pregnancies, 335 stillbirths were reported. Stillbirth increased significantly among women that lived in the hills ecological zones (aRR 1.38, 95% CI 1.02, 1.87) or in the mountains ecological zones (aRR 1.71, 95% CI 1.10, 2.66). Women with no schooling (aRR 1.72, 95% CI 1.10, 2.69), women with primary education (aRR 1.81, 95% CI 1.11, 2.97); open defecation (aRR 1.48, 95% CI 1.00, 2.18), and those whose major occupation was agriculture (aRR 1.80, 95% CI 1.16, 2.78) are more likely to report higher stillbirth.

**Conclusions:**

Low levels of education, ecological zones and open defecation were found to be strong predictors of stillbirth. Access to antenatal care services and skilled birth attendants for women in the mountainous and hilly ecological zones of Nepal is needed to further reduce stillbirth and improved services should also focus on women with low levels of education.

## Introduction

Stillbirth refers to the birth of a baby with no signs of life at or after 28 weeks' gestation[1]. Globally, stillbirth is a major public health problem, with more than 2.7 million stillbirths occurring annually; of these, 98% are from developing countries[2]. Sub-Sahara Africa and South Asia account for the highest numbers of stillbirth[3]. The long-lasting impact of stillbirth remains a large burden for parents, families, policy makers and public health practitioners[4]. Evidence has shown that stillbirth is associated with physical and psychological morbidity, and remains a significant source of cost for the affected family and community [2, 5, 6]. Despite the huge burden of stillbirth on families and global health, progress made in low-middle-income countries to reduce stillbirth is considerably slower than the decline in child mortality[3].

Stillbirth rates vary within and between countries; with economically disadvantaged communities having higher rates compared to their economically well-off counterparts [3, 7]. In developing countries, the major risk factors for stillbirth include advanced maternal age, maternal educational status, infections, fetal development, environmental hazards, diabetes, malaria and umbilical cord complications [8–11]. Recent studies from developed countries (such as the United Kingdom and Sweden) have also reported that psychological issues are associated with higher stillbirth rates [12, 13].

The major causes and predictors of stillbirth in South Asia are not well understood because of the huge variation in data availability and quality that underestimates the true number of stillbirths[5]. Recent case-control studies [14, 15] conducted in Nepal found that stillbirth is associated with older maternal age, lower level of maternal education, coming from the poorest households, inadequate antenatal care and antepartum haemorrhage. Similarly, a verbal autopsy study conducted in Nepal revealed that obstetric complications which included prolonged labour, antepartum haemorrhage and pregnancy induced hypertension were associated with stillbirth[16]. A community-based study from a rural area of Nepal found that a history of prior child loss, maternal age above 30 years and low socio-economic status were associated with higher stillbirth rates[17].

The 2011 Lancet series on stillbirths suggested that for better estimation and intervention, the epidemiology of stillbirths should be at a country level instead of at the regional level because of the regional variations[4]. A major limitation of these Nepalese studies is that the findings cannot be used to inform initiatives and policy responses at the national level because the samples do not represent geographically diverse population across the country. Hence, the aim of this study was to provide nationally representative evidence on the socio-economic predictors associated with stillbirths in Nepal, using pooled data from the 2001, 2006 and 2011 Nepal Demographic and Health Surveys (NDHS). Findings from this study would enable public health professionals to inform different policies and programmes to reduce stillbirth, with subsequent improvements in maternal and newborn outcomes in Nepal.

## Methods

### Data sources

The NDHS is nationally representative, collected by the Nepalese Ministry of Health and Population, in collaboration with New ERA and ICF International, USA using a multi-stage cluster sampling design. Data on fertility, mortality, family planning, and important aspects of nutrition, health, and health services were collected for the years 2001, 2006 and 2011 using standard model questionnaires designed for, and widely used in developing countries[18–20]. For the 2001 NDHS, 8726 women aged 15–49 were interviewed, of these 7089 pregnancies were 7+ months’ gestation. Similarly in the 2006 NDHS, 10,793 women aged 15–49 years were interviewed. Of these, 5921 pregnancies were 7+ months’ gestation. In the 2011 NDHS, 12,674 women aged 15–49 years were interviewed; of which 5376 reported pregnancies 7+ months’ gestation. A total sample of 18,386 pregnancies 7+ months’ gestation five year prior each survey was included in the final analysis. For the year 2006 and 2011, pregnancies were identified using calendar information such as pregnancy outcomes and duration of pregnancy; whereas for the year 2001, pregnancies were identified using information such as pregnancy history index, and outcome and duration of pregnancy. Further detail of the survey methodology, sampling procedure, and questionnaires are reported elsewhere[18–20]. In all surveys, the response was more than 98%.

### Study outcome

The outcome variable was stillbirth, defined as the birth of a baby with no signs of life at or after 28 weeks' gestation[1]. The outcome was recorded as a binary variable in the data set coded as 1 for ‘stillbirth’ and 0 for ‘Alive at birth’. Information on stillbirth was obtained using reproductive calendar (for 2006 and 2011 NDHS); and pregnancy history and outcome of pregnancies (for 2001 NDHS).

### Exploratory variables

The exploratory variables selected for this study were based on previous studies from developing countries [8, 14, 16, 21–23] and the information available in the pooled data sets.

**Fig 1:**
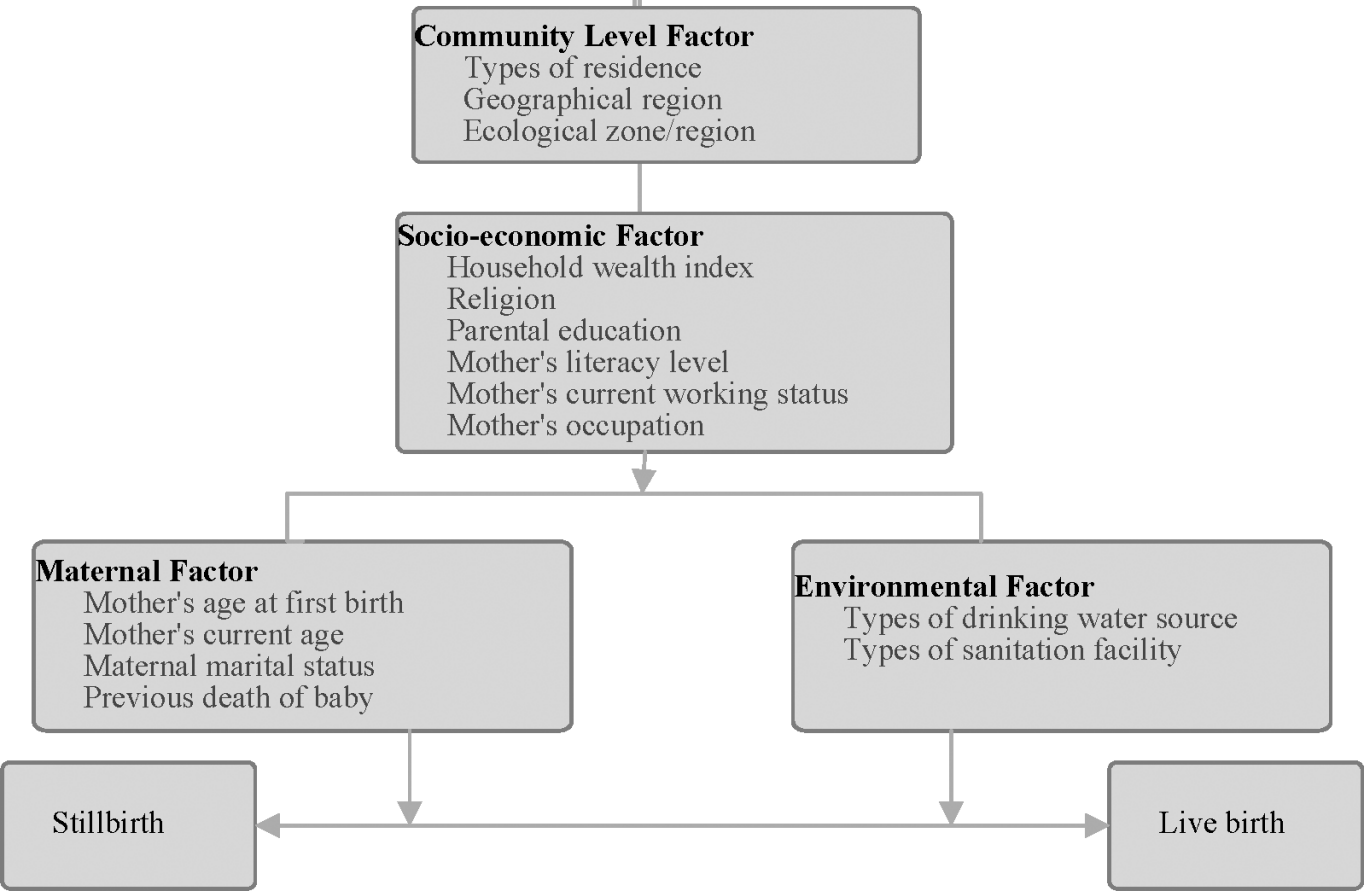
Conceptual framework of socio-economic predictors of stillbirth in Nepal.

Fig 1 presented the modified Mosley and Chen [24] conceptual framework which comprise four groups of variables used in this study: community level factors, socio-economic level factors, maternal factor and environmental factors. The community level factors assessed included ecological zone (terai, hill and mountain), Geographical region (Eastern, Central, Western, Mid-Western and Far-Western) and place of residence (rural or urban). The socio-economic level factors considered were maternal education, literacy level, occupation (categorised as not working or working in agricultural or working in non-agricultural sector), paternal education, mother’s current work status and household wealth index. The household wealth index measures the economic status of the household. As a measure of household wealth index, we pooled the wealth index factor scores in each of the three original Individual Recode data files as calculated by original DHS. The pooled original household wealth index factor scores were then categorised into three: the bottom, 40% of households was referred to as poor households, the next 40% as the middle households and the top 20% as rich households[25].

Maternal factors encompass maternal age at first birth, previous death of a baby, mother’s current age and maternal marital status. We also considered environmental factors consisting of drinking water source and types of sanitation facility for each household classified based on the WHO and UNICEF Joint Monitoring Program (JMP) guidelines[26]. Based on JMP guidelines, we categorized sources of drinking water as: piped water on premises (piped water system into dwelling), other improved drinking water sources (neighbours tap or tubewell, tubewell or borehole in yard, stone tap, protected well and rainwater), unimproved water sources (unprotected well in house, unprotected public or neighbour’s well, unprotected spring, bottled water and water from tanker or truck) and surface drinking water sources (river, stream, pond, lake, dam, canal, or irrigation water). Similarly, we categorized types of sanitation facility as improved and unimproved. Improved sanitation facilities included (households with flush toilet, ventilated or improved pit latrine, pit latrine with slab and composting toilet). Unimproved sanitation facilities were traditional pit toilet, pit latrine without slab or open pit and bucket toilet and open defecation (bush or field for defecation).

### Statistical analysis

Frequency tabulations were first conducted to describe the frequency and relative frequency of all potential confounding factors. This was followed by calculating the stillbirth rate and 95% confidence interval, using ‘the number of stillbirths divided by the number of live births multiplied by 1,000’.

Generalized linear latent and mixed models (GLLAM) with the log link and binomial family[27] that adjusted for cluster and survey weights were used to identify those socio-economic predictors associated with stillbirth. A staged modelling technique[28] was adopted. Community-level factors were first entered into the baseline multivariable model with manual backward elimination process to keep statistically significant variables with p-value <0.05 (model 1). Second, socio-economic factors were added into community-level factors associated with outcome variable and those factors with p-values < 0.05 were retained after backward elimination process was conducted (model 2). Third, maternal factors were added into model 2. After applying similar approach as above, variables with p-values < 0.05 were retained in the next model (model 3). In the final stage, environmental factors were assessed with a list of significant variables from model 3. Variables with p-values < 0.05 were retained in the final model (model 4). Only those factors significantly associated with stillbirth at a 5% significance level in the final model were reported in the study. In the final model, collinearity was tested and reported. The analysis was restricted to five years preceding each of the survey.

A total of 57 missing values were excluded from the multivariate analysis, and GLLAM estimates were translated to relative risk and 95% confidence interval. All analyses were performed using STATA statistical software, version 14.1 (Stata Corporation, College Station, TX, USA) with ‘Svy’ commands to allow for adjustments for sampling weights and cluster sampling design.

### Ethics

The DHS project obtained ethical approval from the Nepal Health Research Council-Kathmandu. The first author communicated with MEASURE DHS/ ICF International and permission was granted to download and use the data for his doctoral dissertation with the School of Science and Health at Western Sydney University, Australia.

## Results

### Basic characteristics of the study participants

The majority of mothers who reported higher rates of stillbirth were from rural and mountainous areas, poor households, parents with low levels of education, households with unimproved sources of drinking water and unimproved toilet facilities (sanitation) (Table 1). We also noted that mothers whose major occupation was agriculture had more stillbirths compared to those mothers who worked in non-agricultural sectors.

**Table 1:** Characteristics of study population as weighted counts and stillbirth, with rates with 95% confidence interval in Nepal: 2001, 2006 and 2011 (N = 18249).

Study variables	N	Stillbirth (n)	Rate (95% CI)	
**Type of residence**				
Urban	1656	27		17(10.3 to 22.8)
Rural	16593	308		19(16.8 to 21.0)
**Ecological zone**				
Terai	9358	154		17(14.1 to 19.4)
Hill	7405	141		19(16.2 to 22.6)
Mountain	1487	41		28(19.7 to 37.1)
**Geographical region**				
Eastern	4154	75		18(14.2 to 22.5)
Central	5936	98		17(13.5 to 20.1)
Western	3363	62		19(14.1 to 23.5)
Mid-western	2596	53		21(15.2 to 26.5)
Far-western	2201	47		22(15.6 to 28.1)
**Wealth index**				
Rich	3615	48		13(9.5 to 17.0)
Middle	7595	163		21(18.2 to 24.8)
Poor	7040	124		18(14.5 to 20.7)
**Religion**				
Hindu	15288	16		12(6.0 to 17.4)
Buddhist	1385	288		19(17.0 to 21.4)
Others	1576	32		21(13.5 to 27.9)
**Mother education**				
Secondary or higher	3833	46		12(8.6 to 15.7)
Primary	3122	63		21(15.5 to 25.7)
No education	11295	227		21(17.8 to 23.2)
**Mother's literacy level (N = 18228)**				
Can read	8096	126		16(13.0 to 18.6)
Cannot read	10133	210		21(18.3 to 24.0)
**Father's education**				
Secondary or higher	3386	40		12(8.2 to 15.7)
Primary	6463	113		18(14.5 to 21.1)
No schooling	8400	182		22(18.9 to 25.4)
**Mother current working status**				
Not working	5470	72		13(10.3 to16.4)
Currently Working	12779	263		21(18.5 to 23.6)
**Mother occupation (N = 18247)**				
Not working	3834	46		12(8.6 to 15.7)
Agriculture	12849	271		22(19.0 to 24.1)
Non- agriculture	1565	18		12(6.3 to 17.0)
**Mother’s age at first birth in years (N = 18191)**				
<18	8043	106		13(10.7 to 19.0)
19–24	9036	148		16(13.7 to19.0)
25+	1112	23		21(12.2 to29.1)
**Mother current age**				
20–29	11643	201		18(15.1 to 20.0)
<20	1198	22		19(10.9 to 26.5)
30–39	4527	94		21(16.9 to 25.5)
40–49	882	17		20(10.3 to 29.0)
**Maternal marital status**				
Currently married	18069	332		19(16.7 to 20.7)
Not currently married	180	4		23(0.5 to 45.0)
**Previous death of baby**				
No	13457	254		19(16.9 to 21.6)
Yes	4793	82		17(13.6 to 21.2)
**Types of drinking water source (N = 17092)**				
Piped water on premises	1843	26		14(8.7 to 19.5)
Other improved drinking water sources	12012	205		17(14.7 t019.4)
Unimproved drinking water sources	1203	26		22(13.3 to29.9)
Surface drinking water sources	2035	53		26(19.0 to33.1)
**Types of sanitation facility (N = 17093)**				
Improved sanitation facilities	4337	50		12(8.3 to14.7)
Unimproved sanitation facilities	1859	36		19(13.0 to 25.7)
Open defecation	10898	224		21(17.9 to23.2)

**Fig 2:**
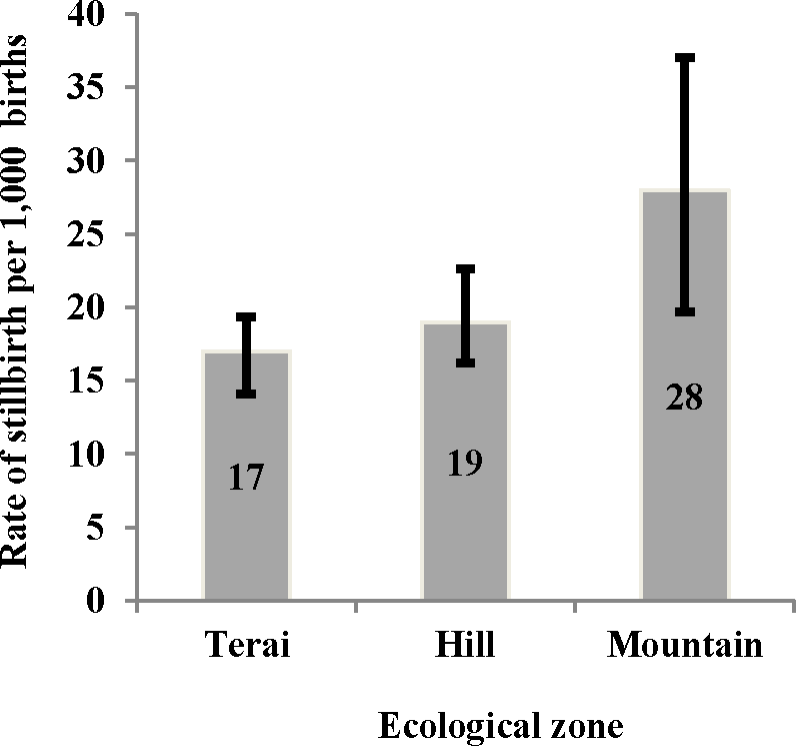
Pooled stillbirth rate by ecological zone in Nepal, 2001–2011.

The prevalence of stillbirth across three ecological zones indicates that the rate was 28 per 1000 amongst mothers who resided in the mountains whereas this rate was 17 per 1000 in the terai, and 19 per 1000 in the hills (Fig 2).

### Predictors of stillbirth

The univariate analyses revealed that ecological zone (mountains or hills); religion (Muslim, Christian and others); mother’s literacy (illiterate); parental level of education (primary education or no schooling), currently not working mothers, mother’s whose major occupation was agriculture, mother’s age (25 years and above at the time of the first birth), types of drinking water source (surface drinking water sources) and types of sanitation facility (unimproved sanitation facility or open defecation) were all significantly associated with higher stillbirth (Table 2).

**Table 2:** Crude and adjusted Relative Risk (RR) for socio-demographic predictors of stillbirths in Nepal, 2001–2011 (N = 18249).

Study variables	Unadjusted		Adjusted	
	RR (95% CI)	P-value	RR (95% CI)	P-value
**Type of residence**				
Urban	Reference			
Rural	1.07 (0.71, 1.61)	0.738		
**Ecological zone**				
Terai	Reference		Reference	
Hill	1.17(0.90, 1.51)	0.241	1.37(1.02, 1.84)	0.036
Mountain	1.71(1.16, 2.52)	0.006	1.68(1.09, 2.59)	0.018
**Geographical region**				
Eastern	Reference			
Central	0.88(0.63, 1.25)	0.478		
Western	1.01(0.69, 1.48)	0.956		
Mid-western	1.11(0.74, 1.65)	0.621		
Far-western	1.17(0.77, 1.77)	0.459		
**Wealth index**				
Rich	Reference			
Middle	1.56(1.12, 2.17)	0.009		
Poor	1.28(0.91, 1.80)	0.156		
**Religion**				
Buddhist	Reference		Reference	
Hindu	1.64(0.97, 2.76)	0.062	2.19(1.19, 4.04)	0.012
Others including Muslim and Christian	1.87(1.00, 3.50)	0.05	2.68(1.29, 5.58)	0.008
**Mother education**				
Secondary or higher	Reference		Reference	
Primary	1.67(1.14, 2.45)	0.009	1.80(1.10, 2.93)	0.019
No schooling	1.65(1.20, 2.28)	0.002	1.71(1.09, 2.66)	0.019
**Mother's literacy level**				
Can read part or whole of the sentence	Reference			
Cannot read	1.31(1.05, 1.65)	0.018		
**Father's education**				
Secondary or higher	Reference			
Primary	1.47(1.02, 2.12)	0.036		
No schooling	1.83(1.30, 2.59)	0.001		
**Mother current working status**				
Not working	Reference			
Currently working	1.54(1.18, 2.01)	0.002		
**Mother occupation**				
Not working	Reference		Reference	
Agriculture	1.70(1.24, 2.34)	0.001	1.78(1.16, 2.75)	0.009
Non- Agriculture	0.99(0.57, 1.70)	0.964	1.38(0.72, 2.63)	0.328
**Mother’s age at first birth**				
<18	Reference		Reference	
19–24	1.23(0.95, 1.58)	0.109	1.20(0.93, 1.56)	0.186
>25	1.59(1.00, 2.50)	0.046	1.77(1.12, 2.82)	0.015
**Mother current age**				
<20	Reference			
20–29	1.10(0.71, 1.70)	0.675		
30–39	1.18(0.92, 1.51)	0.180		
40–49	1.15(0.70, 1.88)	0.590		
**Maternal marital status**				
Currently married	Reference			
Not currently married	1.20(0.44, 3.28)	0.720		
**Previous death of baby**				
No	Reference			
Yes	0.88(0.69, 1.14)	0.332		
**Types of drinking water source**				
Piped water on premises	Reference			
Other improved drinking water sources	1,21(0.80, 1.84)	0.371		
Unimproved drinking water sources	1.53(0.88, 2.65)	0.135		
Surface drinking water sources	1.81(1.12, 2.94)	0.016		
**Types of sanitation facility**				
Improved sanitation facilities	Reference		Reference	
Unimproved sanitation facilities	1.57(1.01, 2.43)	0.044	1.10(0.67, 1.82)	0.697
Open defecation	1.76(1.29, 2.41)	<0.001	1.47(1.00, 2.16)	0.049

Multivariable analysis revealed that factors associated with stillbirth were mothers in the age bracket (>25years), mothers who lived in mountains or hills, mothers whose religion was Hindu, Muslim, Christian and others, mothers who had no schooling or only primary level of education. Further we found that mothers whose major occupation was agriculture and those who used open defecation reported higher stillbirth.

In the final model, we removed maternal education level and replaced it with father’s education level; the result indicated that stillbirth increased significantly among fathers with no schooling (aRR 1.71, 95% CI 1.10, 2.64).

## Discussion

This study reports the predictors associated with stillbirths in Nepal by using pooling the three most recent Nepal demographic and Health survey and found that maternal age (25 years and over), low levels of education, sanitation and ecological zones were predictors for stillbirth. Additionally, when mother education was replaced by father education in the final model, father with no education reported significantly higher stillbirth. This current study provides an evidence-base that could be used to inform the design of effective interventions, policies and programmes aimed at health professionals and individuals recognising stillbirths.

Primiparity is an established risk factor for stillbirth in both high and low income countries[29], and our results also found this association. This study demonstrated that mothers aged 25 years and above at the time of their first birth were more likely to experience stillbirth. This finding was supported by case-control studies conducted in Nepal, Bangladesh and Canada, which indicated that older mothers (35 years and above) significantly reported higher stillbirth than younger mothers [14, 22, 30]. Similarly, a hospital-based study conducted in Nigeria also revealed that mothers aged 35 years or older were significantly more likely to report higher rate of stillbirths[31]. Studies conducted in high-income countries showed a significant relationship between stillbirth and maternal age [7, 32–35]. Higher stillbirth rate in older women has been attributed to the increase likelihood of congenital anomalies, chronic hypertension, placenta praevia, uterine rupture, and breech deliveries in older mothers which may contribute to an increased fetal death [36–39]. Studies have also shown that advanced maternal age has been associated with an increased risk of abnormal chromosomes, and or decreasing uterine and hormonal function[40, 41].

Parental education is considered as one of the important determinants of health. Previous studies from Pakistan and Bangladesh reported that education could increase the uptake of health service utilization [42, 43] with subsequent reduction in stillbirth. This study found that women with only primary level of education or no schooling had higher risk of stillbirth compared to those who had secondary or higher levels of education. There are very few studies from developing countries that have examined the relationship between maternal education and stillbirth. A study conducted in a province of Thailand revealed women who had low levels of education were at a higher risk of having stillbirths[44]. This finding is also consistent with previous studies from Canada and Denmark, which found that lower level of maternal education was associated with higher risk of stillbirths [45–47]. Plausible reasons for this finding may be that educated mothers are more likely to practice healthy behaviours, including health seeking, which may contribute to reducing their risk of stillbirth compared to mothers with no education. Likewise, fathers with no schooling were also associated with higher risk of stillbirth.

Our study demonstrated that the risk of stillbirth was significantly higher among women who worked in an agricultural sector, similar to a finding from a hospital-based case-control study conducted in the Nguyen province of Vietnam[44]. Our finding of higher risk of stillbirth among mothers residing in the high altitude mountains or hills was similar to a retrospective births record obtained from four regional centres in Peru, which indicated that after controlling for potential confounding factors, mothers who lived in high altitude (greater than 3000 meters) were significantly more likely to report higher stillbirths than those mothers that lived in low altitude [48]. Whether our finding is related to altitude or access to antenatal and birth service is unknown due to the limitations of the DHS data. However, literature has shown that management of pregnancy complications through quality antenatal care[49] and provision of skilled birth attendance around labour time[4] help to prevent stillbirth. Based on these evidences, it can be argued that the focused antenatal care as well as targeted skilled birth attendance for women residing in the mountainous region would help to reduce the number of stillbirth.

Our study also found an association between stillbirth and mothers religious affiliation. Mothers whose religion was Hindu and others including Muslim and Christian reported significantly higher stillbirth compared to those mothers whose religion was Buddhist. Analysis [50] conducted in India using the National Family Health Survey (NFH) reported differences in child mortality based on religious affiliation. In Nepal access and utilization of birthing services differs by religious affiliation and this may contribute to the increased stillbirth in some religious groups[51].

Unimproved water and sanitation contributes 0.9% to the global Disability Adjusted Live Years [52]. It is not surprising that women who reported open defecation were at greater risk of stillbirth compared to mothers who reported improved sanitation facilities; similar with the finding from a population-based prospective cohort study conducted in India that revealed open defecation among pregnant women was associated with adverse pregnancy outcomes[53].

The study has a number of strengths. Firstly, the analysis was based on nationally representative data (NDHS); thus, estimates from this study are generalizable to the Nepalese population and can inform national policies and initiatives in Nepal. Secondly, the response to the surveys was high (>98%), reducing a likely chance of selection bias from the observed findings. Thirdly, measurement bias is unlikely to affect the observed results as the data were collected using a standardised questionnaire developed for developing countries including Nepal[18–20]. It is however retrospective data, and there may be some bias in reporting stillbirth. Despite these advantages, this study is limited in a number of ways. Firstly, the diagnosis of stillbirth was based on self-report from mother and is subject to recall and misclassification bias. Secondly, formal verbal autopsies were not conducted on stillbirths. Finally, no information on health services factors or other factors such as tobacco, gestational diabetes and genetic abnormality that may have been associated with stillbirth were included in the NDHS data.

## Policy implications

To close the equity gaps, community-based interventions need to be formulated and implemented in order to improve maternal and child health in Nepal. At the individual level intervention, uptake and quality of antenatal care should be encouraged among mothers from low socio-economic group and those mothers from hilly and mountainous ecological zones. At the community level intervention, increase awareness and access to basic and emergency obstetric care to women from hilly and mountainous ecological zones. These interventions will improve prevention strategies that could have massive and far-reaching improvement on Nepalese mothers and children in order for the country to accelerate progress towards achievement of ending preventable stillbirths by 2035[54].

## Conclusions

Our findings suggest that antenatal care service should be targeted to women from low socioeconomic status and those who lived in the mountainous ecological zone in order for Nepal to further reduce the rate of stillbirth to a target of 12 stillbirths per 1000 births by the year 2030.
